# Combinatorial optimization of pathway, process and media for the production of p‐coumaric acid by *Saccharomyces cerevisiae*

**DOI:** 10.1111/1751-7915.14424

**Published:** 2024-03-25

**Authors:** Sara Moreno‐Paz, Rianne van der Hoek, Elif Eliana, Vitor A. P. Martins dos Santos, Joep Schmitz, Maria Suarez‐Diez

**Affiliations:** ^1^ Laboratory of Systems and Synthetic Biology Wageningen University & Research Wageningen The Netherlands; ^2^ Department of Science and Research–dsm‐firmenich, Science & Research Delft The Netherlands; ^3^ Bioprocess Engineering Group Wageningen University & Research Wageningen The Netherlands

## Abstract

Microbial cell factories are instrumental in transitioning towards a sustainable bio‐based economy, offering alternatives to conventional chemical processes. However, fulfilling their potential requires simultaneous screening for optimal media composition, process and genetic factors, acknowledging the complex interplay between the organism's genotype and its environment. This study employs statistical design of experiments to systematically explore these relationships and optimize the production of p‐coumaric acid (pCA) in *Saccharomyces cerevisiae*. Two rounds of fractional factorial designs were used to identify factors with a significant effect on pCA production, which resulted in a 168‐fold variation in pCA titre. Moreover, a significant interaction between the culture temperature and expression of ARO4 highlighted the importance of simultaneous process and strain optimization. The presented approach leverages the strengths of experimental design and statistical analysis and could be systematically applied during strain and bioprocess design efforts to unlock the full potential of microbial cell factories.

## Graphical abstract

We used statistical design of experiments to simultaneously optimize gene expression, media composition and culture conditions for the production of p‐coumaric acid by *Saccharomyces cerevisiae*. This study exemplifies the importance of simultaneous optimization and the interaction between genetic and environmental factors.

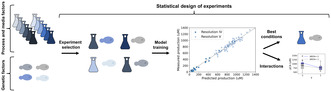

## INTRODUCTION

Microbial cell factories play a pivotal role in driving the transition towards a bio‐based economy, being a sustainable alternative to traditional chemical processes (Kim et al., 2023). Microorganisms can efficiently transform raw materials into valuable products. However, to unlock their potential for biotransformation in an economically feasible manner, it is essential to optimize production pathways and bioprocesses (de Lorenzo & Couto, 2019).

Pathways can be optimized sequentially by tuning individual genetic factors in isolation. However, this does not capture the complex interplay between different genetic elements and the products they code for. It is hence desirable to perform combinatorial pathway optimization, which is based on the simultaneous optimization of multiple genetic factors and facilitates the identification of complex interactions (Gilman et al., 2021; Jeschek et al., 2017). Moreover, the overall performance of the microbial cell factory is not only determined by its genotype but is also influenced by the production conditions, as factors such as media nutrients, pH, cultivation temperature and aeration influence cell physiology and metabolism. Strains are usually optimized holding the environmental conditions constant and only the most promising strain advances to the bioprocess optimization stage (Carbonell et al., 2018; Song et al., 2017). However, this approach might ignore genetic designs that, although inferior in standard laboratory conditions, have bigger potential when the media and bioprocess are optimized (Zhou et al., 2015). Only by simultaneously screening for optimal media composition and genetic factors, the dynamic interplay between the organism's genotype and the environment in which it operates can be considered (Brown et al., 2018; Zhou et al., 2015).

Combinatorial optimization of strains, media and process parameters, however, requires exponentially increasing resources. Statistical design of experiments (DoE) allows a structured exploration of the relationships between experimental variables (factors) and the measured response. Full factorial designs are a type of DoE design that tests all possible combinations of factor levels, characterizing factor effects and allowing the estimation of interactions. The number of experiments to be performed depends on the number of genetic and environmental factors to be tested (e.g. expression of a gene, temperature) and the number of levels per factor (e.g. low, medium and strong gene expression, 20, 25, 20 and 35°C) according to $\prod_{i=1}^{F}L_{i}$, where *F* is the number of factors and *L*_*i*_ is the number of levels of factor *i*. In these designs, the effect of a factor is not only estimated considering replicate experiments but also all the experiments where the given factor is constant regardless of other factor's levels. This property can be leveraged in fractional factorial designs that reduce the number of experiments to perform while maximizing the information gain. This is achieved by performing experiments that preserve orthogonality in the desired factors, i.e. ensuring that the effect of a factor is not confounded by planned changes in other factors. The generated data is fitted to a linear model so main effects (MEs), representing the impact of not‐confounded factors on the response, are identified. Similarly, the so‐called two‐factor interactions (2FIs) that occur when the effect of a factor on the response changes based on the level of another factor, can also be estimated (Lawson, 2014).

Although decreasing the number of experiments still ensures the identification of not‐confounded effects, information regarding confounded factors or interactions is lost (Lawson, 2014). For example, resolution IV designs, a type of fractional design, allows the identification of MEs but confounds 2FIs among each other. Therefore, these designs can be used to report if 2FIs are important but cannot clarify which 2FIs have a significant effect on the response. Fractional designs with lower resolution, such as resolution III designs, require fewer experiments but confound ME with 2FI. These designs should therefore only be used when interactions among factors are not expected, rarely true for biological systems. Alternatively, designs with higher resolution ensure the identification of interactions at the expense of a higher experimental workload.

We used the production of p‐coumaric acid (pCA) by *Saccharomyces cerevisiae* as an example of DoE‐aided combinatorial pathway, media and process optimization. pCA can be produced from phenylalanine (Phe), an aromatic amino acid produced within the shikimate pathway, and is a precursor for a wide array of biologically relevant molecules such as pharmaceuticals, flavours, fragrances and cosmetics (Liu et al., 2019). Although pCA production has been independently optimized at the strain and bioprocess levels (Combes et al., 2021; Koopman et al., 2012; Liu et al., 2019), we show the interplay between genetic and environmental factors highlighting the importance of simultaneous process and strain optimization.

## EXPERIMENTAL PROCEDURES

### Strain construction

Promoter, terminator and open reading frames (ORFs) sequences from *aro4*, *aroL*, *aro7*, *pal1*, *c4h* and *cpr* codon optimized for *S. cerevisiae* were obtained from Moreno‐Paz et al. (2023) (Table S1). Twelve cassettes formed by combinations of promoter, ORF and terminator (Table S1) were assembled via Golden Gate into a backbone plasmid containing a 50 bp homologous connector sequence to facilitate in vivo recombination of the gene cluster (Verwaal et al., 2018). Golden Gate products were transformed into chemically competent *Escherichia coli* DH10B cells, plasmids were isolated and cassettes were confirmed by PCR.

Strains were constructed as described in Moreno‐Paz et al. (2023). In short, a host strain with Cas9 integrated in the non‐coding region between YOR071c and YOR070c in chromosome 15 was transformed with a linear guide RNA targeting the AEHG01000256.1 locus (NCBI, 2024) (210 ng/kb), equimolar cassettes for the required designs (Table 1; Table S2) (100–300 ng/kb) and linear backbone fragments (35 ng/kb) following the LiAc/ssDNA/PEG method (Gietz et al., 1995). The connector sequences on the cassettes facilitate in vivo recombination of a cluster of genes in the genome (Verwaal et al., 2018). Transformants were plated on Qtray (NUNC) with 48‐divider (Genetix) containing YEPhD agar medium and selection agent. Colonies appeared on the plate after 3 days of incubation at 30°C. Single colonies were picked with Qpix 420 (Molecular Devices) into a 96‐well plate containing YEPhD agar medium and selection agent and regrown for 3 days at 30°C. Colonies were confirmed using whole genome sequencing as described in Moreno‐Paz et al. and correct strains were stored in 10% glycerol at −80°C.

**TABLE 1:** Structure of the gene clusters.

Gene cluster	ARO4	AROL	ARO7	PAL1	C4H	CPR1
1	TDH3	TEF1	ACT1	RPS9A	CHO1	CCW12
2	TDH3	TEF1	ACT1	VMA6	PXR1	CCW12
3	TDH3	TEF1	PFY1	RPS9A	CHO1	CCW12
4	TDH3	TEF1	PFY1	VMA6	PXR1	CCW12
5	TDH3	RPL28	ACT1	RPS9A	CHO1	CCW12
6	TDH3	RPL28	ACT1	VMA6	PXR1	CCW12
7	TDH3	RPL28	PFY1	RPS9A	CHO1	CCW12
8	TDH3	RPL28	PFY1	VMA6	PXR1	CCW12
9	MYO4	TEF1	ACT1	RPS9A	CHO1	CCW12
10	MYO4	TEF1	ACT1	VMA6	PXR1	CCW12
11	MYO4	TEF1	PFY1	RPS9A	CHO1	CCW12
12	MYO4	TEF1	PFY1	VMA6	PXR1	CCW12
13	MYO4	RPL28	ACT1	RPS9A	CHO1	CCW12
14	MYO4	RPL28	ACT1	VMA6	PXR1	CCW12
15	MYO4	RPL28	PFY1	RPS9A	CHO1	CCW12
16	MYO4	RPL28	PFY1	VMA6	PXR1	CCW12

*Note*: Cell values indicate the promoter used for each gene. Promoters were selected from (Moreno‐Paz et al., 2023).

### pCA production experiments

Single colonies were grown in 10 mL YPDA media (Takara) in 50 mL tubes for 24 h. Cultures were washed and inoculated in minimal media at starting optical densities (ODs) of 0.3 or 0.6 according to the experimental design. Minimal media contained 20 g/L glucose (Acros Organics) and 1.7 g/L yeast nitrogen base without amino acids or ammonium sulfate (BD Difco). A 60.5 mM nitrogen concentration in the media was obtained with 4 g/L ammonium sulfate (Acros Organics) or 1.82 g/L urea (Acros Organics). When required, media was buffered at a pH of 7 using 126 mM Na_2_HPO_4_ (Acros Organics) and 18 mM citric acid (Sigma‐Aldrich) (Prins & Billerbeck, 2021) and/or supplemented with 5 mM Phe (Sigma‐Aldrich) and/or 5 mM Glu (Sigma‐Aldrich). Cells were grown for 48 h at the required temperature and agitation speed in 50 mL mini‐bioreactor tubes (Corning) in an Innova 44 incubator (New Brunswick Scientific). At the end of the cultivation samples for OD measurements and pCA quantification were taken.

### pCA quantification

For pCA quantification, 400 μL of culture was mixed with 800 μL acetonitrile (Thermo Scientific) and centrifuged for 10 min at 4000*g*. The acetonitrile phase was used for analysis using high‐performance liquid chromatography on a Shimadzu LC2030C Plus 2 machine equipped with a Poroshell 120EC‐C18 column (250 × 4.6 mm, Agilent) and a UV/vis detector. Mobile phase was used at a rate of 1 mL/min and was composed of Milli‐Q water (A), 100 mM formic acid (B), and acetonitrile (C) at varying proportions: 77:10:13 (v/v/v) in the first 10 min, 23:10:67 (v/v/v) in the next 9 min, and 77:10:13 (v/v/v) in the last 6 min pCA was detected at a wavelength of 280 nm. Standards were prepared using pCA purchased from Sigma‐Aldrich.

### Experimental design and statistical analysis

The FrF2 function from the FrF2 R package was used for the generation of the designs given the number of factors and the desired resolution (Grömping, 2014). Experimental data were used to train a linear model:

(1) $$y=\beta_{0}+\sum_{i=1}^{i=n}{\mathrm{ME}}_{i}∙F_{i}+\sum_{i=1}^{i=n}\sum_{j=1}^{j=n}2{\mathrm{FI}}_{i:j}∙F_{i}∙F_{j},$$

where *y* represents the pCA concentration; *ME*_*i*_ refers to the main effect of factor *i* (*F*_*i*_) and 2*FI*_*i*:*j*_ refers to the two‐factor interaction between factor *i* and *j*. The number of factors is indicated by *n* indicates the total number of factors.

Ordinary least squares regression minimizing the sum of squared differences between the observed and predicted values was used to estimate the coefficients for each term in the model (*ME*_*i*_, 2*FI*_*i*:*j*_) using the R lm function. Then the summary function was used to obtain the ANOVA table which provides the estimated coefficients and their associated *p*‐values. *p*‐values were corrected using Bonferroni. The adjusted coefficient of determination (Adj *R* ^2^) and the mean absolute error (MAE) were used to assess the model fit to experimental data.

## RESULTS

### Selection of genetic and environmental factors and levels

**FIGURE 1:**
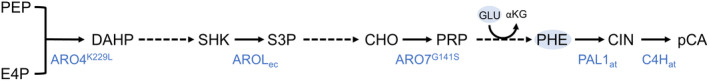
pCA production pathway. Genes whose expression is considered as factor for the design are shown. Glu and Phe are highlighted as they are selected as factors for media optimization. CHO, chorismate; CIN, cinnamate; DAHP, 3‐deoxy‐7‐phosphoheptulonate; E4P, erithrose‐4‐phosphate; GLU, glutamate; pCA, p‐coumaric acid; PEP, phosphoenolpyruvate; PHE, phenylalanine; PRP, phrephenate; S3P, shikimate‐3‐phosphate; SHK, shikimate; αKG, _α_‐ketoglutarate.

The shikimate pathway is tightly regulated and aromatic amino acids exert feedback inhibition on some of its enzymes (Figure 1) (Braus, 1991). Expression of feedback‐resistant variants of ARO4 (ARO4^K229L^) and ARO7 (ARO7^G141S^) are common strategies to increase pCA production (Liu et al., 2019; Rodriguez et al., 2015). Besides, the phosphorylation of shikimate performed by ARO1 has been hypothesized as a rate‐limiting step. Rodriguez et al. reported a beneficial effect of expressing *E. coli* AROL to increase the flux through this reaction (Rodriguez et al., 2015). Therefore, we selected the expression of ARO4^K229L^, ARO7^G141S^ and AROL as genetic factors with the potential to affect pCA production. Each of these factors was evaluated at two levels based on the strength of the promoter‐terminator pair assigned to each gene (Table 2) (Moreno‐Paz et al., 2023).

**TABLE 2:** Factors and levels used for pCA optimization.

Factor	Low‐level (−1)	High‐level (1)
Temperature	20°C	30°C
Agitation	180 rpm	250 rpm
Initial cell density (OD)	0.3	0.6
N‐source	Urea	Ammonium sulfate
pH	Unbuffered	Buffered
Phe	0	5 mM
Glu	0	5 mM
PAL‐C4H promoters	VMA6–PXR1	RPS9A–CHO1
ARO7 promoter	PFY1	ACT1
AROL promoter	RPL28	TEF1
ARO4 promoter	MYO4	TDH3

To produce pCA from Phe, the expression of two heterologous genes is required: phenylalanine ammonia lyase (PAL) and cinnamate 4‐hydroxylase (C4H). Although *S. cerevisiae* contains endogenous cytochrome P450 reductases (CPR), expression of a C4H‐associated CPR is recommended (Jendresen et al., 2015; Koopman et al., 2012; Liu et al., 2019). We expressed *Arabidopsis thaliana* CPR under a constitutive promoter and considered the expression of PAL and C4H as an additional genetic factor for the design. The expression levels of PAL and C4H were evaluated using two promoter‐terminator pairs (Table 2).

Temperature (T), agitation (rpm) and initial cell density (OD) are usual variables tuned during bioprocess optimization and were selected as factors to improve pCA titers (Couto et al., 2017; Duman‐Özdamar et al., 2022; So et al., 2023). Temperature was varied between 30°C, the optimal growth temperature of *S. cerevisiae*, and 20°C, as lower temperatures might improve heterologous pathway expression (Table 2) (So et al., 2023). Agitation and initial OD were varied between 180 and 250 rpm and 0.3–0.6, respectively (Table 2).

Combes et al. (2022) showed that the pH of the media affects pCA production. Acidic pH, below the pKA of pCA (4.65), favours the undissociated form of pCA (pHCA) in the media that diffuses into the cell where it dissociates (pCA^−^), acidifying the cytoplasm and requiring active export at the cost of ATP. Considering this, two factors that influence the pH of the media were selected: the addition of a buffer and the use of different nitrogen sources (Table 2). When ammonium sulfate or urea are used as a nitrogen source, pH below and above the pKA of pCA are expected respectively (Prins & Billerbeck, 2021). Independently of the N‐source used, the citrate phosphate buffer can control the pH of the culture but can negatively impact cell growth (Prins & Billerbeck, 2021).

Media supplementation is an additional common strategy to increase production (Azubuike et al., 2020; Couto et al., 2017; Motta Dos Santos et al., 2016; Song et al., 2017). Phenylalanine is the substrate of PAL, the first enzyme required for the production of pCA and glutamate is the nitrogen donor used during Phe production (Figure 1). Therefore, the additions of these amino acids were considered as additional factors (Table 2).

### Resolution IV design: Impact of individual factors on pCA production

The effect of changing process conditions and media‐related factors on pCA production was evaluated using a resolution IV fractional factorial design. These designs allow the estimation of the MEs of all the factors while confounding 2FIs. They can be used during screening to identify factors with a significant impact on production that can be the focus of later optimization.

**FIGURE 2:**
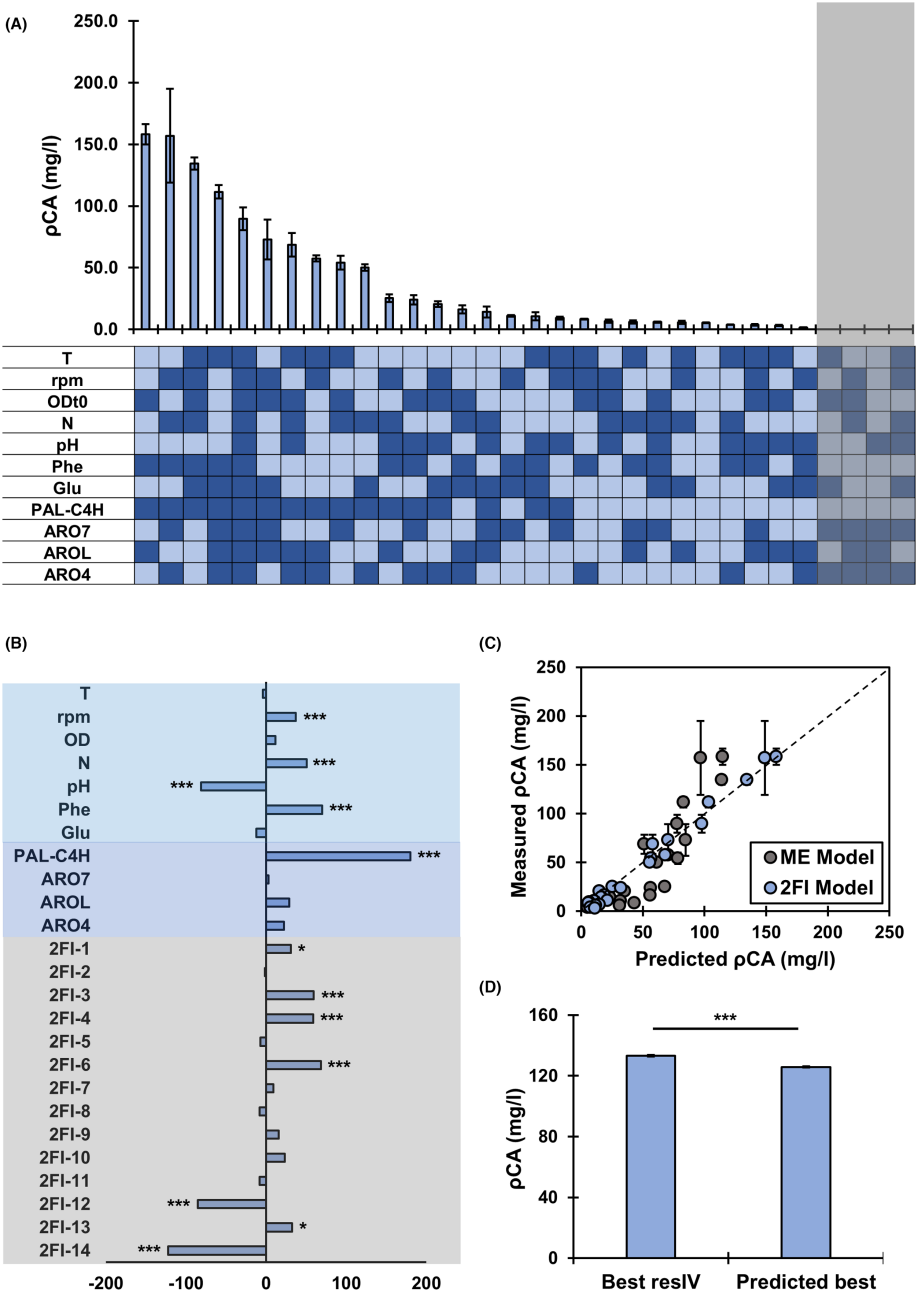
Resolution IV Design. (A) Measured pCA production at different combinations of factors and levels. Light colours indicate low levels (−1) and dark blue indicates high levels (1). The shaded grey area indicates the four experiments that could not be performed. (B) Coefficients of the model including main effects (MEs) and confounded two‐factor interactions (2FIs). ***Indicates corrected *p*‐value ≤0.001, **≤0.01 and *≤0.05. (C) Fit of models including MEs or MEs and 2FIs model to experimental data. ME model: Adj *R* ^2^ = 0.66, MAE = 129; 2FI model: Adj *R* ^2^ = 0.94, MAE = 33. (D) pCA production in validation experiment.

In order to obtain a resolution IV design with 11 factors (Table 2), 32 experiments are required (Figure 2; Table S3). Although traditional applications of DoE use single‐replicate screening, replicates are a necessity to assess biological variation and, for each experiment, pCA production was measured in three independent cultures (Gilman et al., 2021). These experiments involved the construction of 16 strains including all possible combinations of the four selected genetic factors. Each strain is then tested in two different conditions determined by the design. However, strains containing gene clusters 2 and 6 (Table 1) could not be constructed and the effect of not performing four out of the 32 experiments was evaluated. Reducing the number of experiments to 28 did not affect the estimation of the MEs but increased the complexity of the confounding patterns for the 2FI. Considering the goal of the experiment was to determine the MEs, construction of the two additional strains was not required, which accelerated the implementation of the design round. In the 28 experiments performed, pCA production varied two orders of magnitude, from 1.3 to 158.2 mg/L, confirming the impact of the selected factors on production (Figure 2A).

Linear models containing only ME or ME and confounded 2FI were trained. Including 2FI increased the coefficient of determination from 0.66 to 0.94 (Figure 2C). Figure 2B shows the estimated coefficients of the model including MEs and 2FI. An ANOVA was used to determine the significance of each ME and 2FI on pCA production and p‐values were corrected using Bonferroni. All factors but T, OD, Glu and ARO7 had a significant effect on pCA production. Although, seven of the estimated 2FI were also significant, identifying the specific significant 2FI was not possible due to their confounding.

The MEs with the highest impact on performance were the expression strength of PAL and C4H, with a positive regression coefficient. This indicates that a high expression of the heterologous genes for pCA production is essential to obtain high titers. The effect of PAL‐C4H was followed by the negative impact of buffering of the media represented by a negative regression coefficient. Although the addition of a buffer could control the dissociation of pCA (Combes et al., 2022), it negatively affected cell growth and resulted in overall low pCA titers. The third most relevant ME was the addition of Phe, with a positive coefficient that shows the benefit of Phe supplementation on pCA production (Trantas et al., 2009).

Notably, estimated coefficients for 2FI‐6, 2FI‐12 and 2FI‐14 had a similar impact on pCA titre than PAL‐C4H, pH and Phe and the coefficient of determination was significantly improved when 2FIs were considered for pCA production (Figure 2B, C), indicating that a design with a higher resolution that allows the estimation of 2FI is required to optimize pCA production. The importance of 2FI was confirmed in an independent experiment where the best experiment from the resolution IV design was compared to the best‐predicted experiment according to the model's ME. The predicted best experiment showed a small (5.6%) but significant reduction in pCA production, confirming the importance of 2FIs (Figure 2D; Table S4).

### Resolution V design: Identification of relevant 2‐factor interactions

Fractional factorial resolution V designs are required to identify MEs and 2FIs. When 11 factors are considered this results in 128 experiments. In order to decrease the number of experiments, factors with the highest impact on pCA production were fixed: only strains with high expression of PAL‐C4H were considered and unbuffered media supplemented with Phe was used. This reduced the number of factors to 8 and the number of required experiments to 64. Considering the small variation of the resolution IV dataset, duplicates instead of triplicates were used during this round. The top‐producing conditions from the resolution IV experiments were included as control.

**FIGURE 3:**
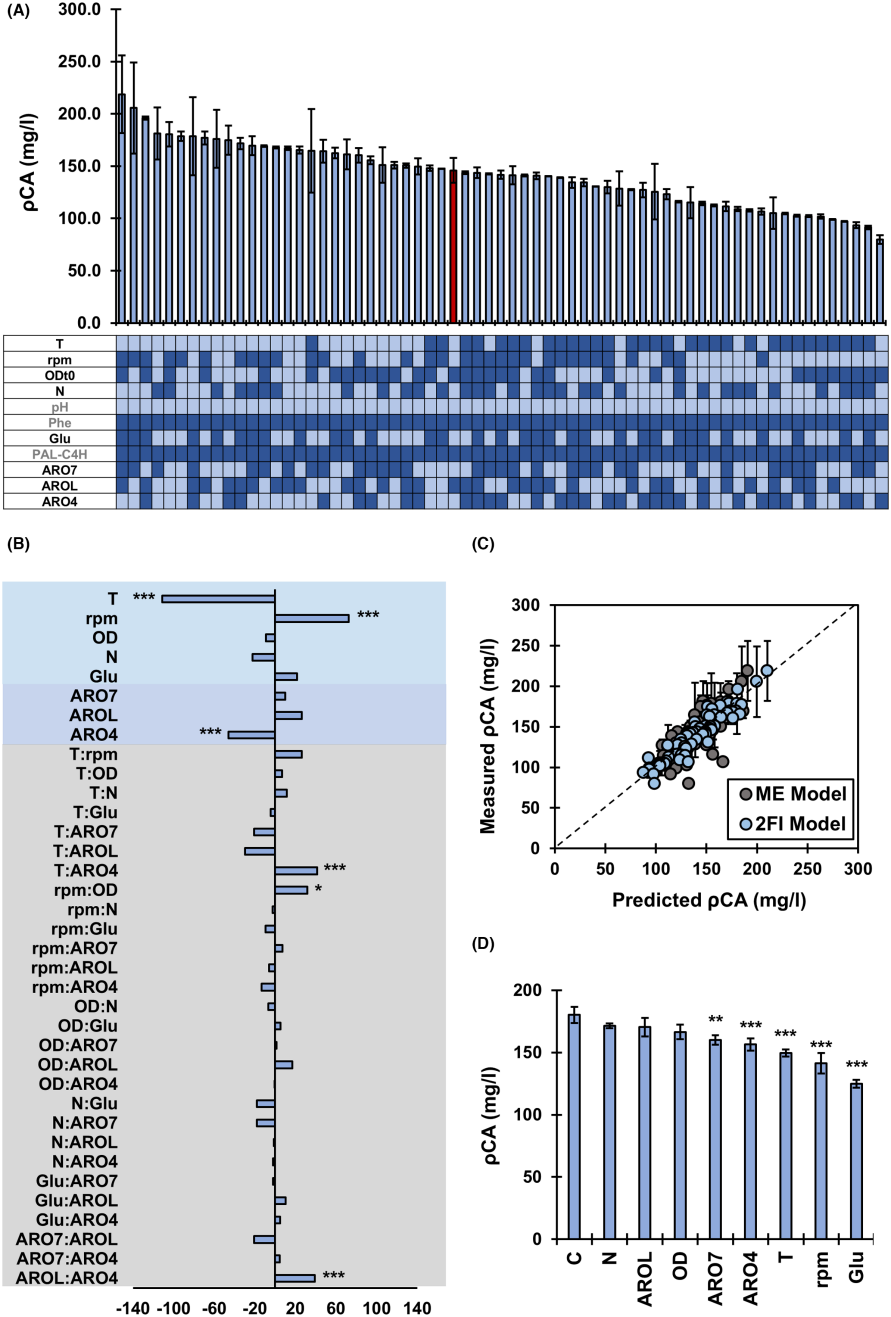
Resolution V Design. (A) Measured pCA production at different combinations of factors and levels. Light blue indicates low levels (−1) and dark blue indicates high levels (1). The red bar is the best‐performing experiment from the resolution IV round. (B) Coefficients of the model including main effects (MEs) and 2‐factor interactions (2FIs). ***Indicates corrected *p*‐value ≤0.001, **≤0.01 and *≤0.05. (C) Fit of models including MEs (ME Model) or MEs and 2FIs (2FI Model) to experimental data. ME model: Adj *R* ^2^ = 0.55, MAE = 82; 2FI model: Adj *R* ^2^ = 0.74, MAE = 51. (D) pCA production in validation experiment.

In the resolution V experiments, pCA production varied from 79.8 mg/L to 218.7 mg/L, improving the maximum production found in the first round by 38% (Figure 3A; Table S5). The use of strains with high expression of PAL‐C4H in unbuffered media supplemented with Phe, increased the minimum production of pCA in this round by 62%, supporting the information provided by the resolution IV model.

Experimental data was used to train new linear models based on MEs or MEs and 2FIs. The model trained with MEs showed a coefficient of determination of 0.55 that increased to 0.74 when 2FIs were considered, highlighting the relevance of 2FI to explain pCA production (Figure 3C).

Temperature and agitation were identified as significant process‐related factors, so low T and high rpm improve pCA production (Figure 3B). ARO4 was the only significant genetic factor, and, in contrast to other reports, low expression of this gene positively affected pCA titers (Figure 3B) (Liu et al., 2019; Rodriguez et al., 2015). Moreover, three significant positive 2FI were found: T:ARO4, rpm:OD and AROL:ARO4 (Figure 3B).

In order to find the optimal strain and conditions for pCA production, the model including MEs and 2FIs was used to predict pCA titers for all strains in all possible media conditions. The use of a strain with high expression of PAL‐C4H, ARO7 and AROL and lower expression of ARO4 in a media supplemented with urea, Phe and Glu incubated at 20°C and 250 rpm with an initial OD of 0.3 was predicted to optimize pCA production. These conditions were met by the top‐producing experiment measured in the resolution V round. To avoid bias towards performed experiments during the estimation of model parameters, a new model was trained excluding data from the top‐producing experiment. When pCA production was predicted, the excluded top producer experiment was also suggested as optimal. Moreover, we evaluated the effect of individually changing each factor to their sub‐optimal level. As expected, these modifications decreased or did not affect pCA production (Figure 3D; Table S6). Changing the initial OD, the nitrogen source or the expression of AROL–all factors with not significant ME–did not significantly change the pCA produced. In contrast, modifying the expression of ARO4, T and rpm, factors with significant ME, negatively impacted pCA production. Although ME related to ARO7 and Glu were insignificant, reducing the expression of ARO7 and omitting Glu supplementation, negatively impacted pCA production, which could be explained by higher‐order factor interactions not included in the model.

**FIGURE 4:**
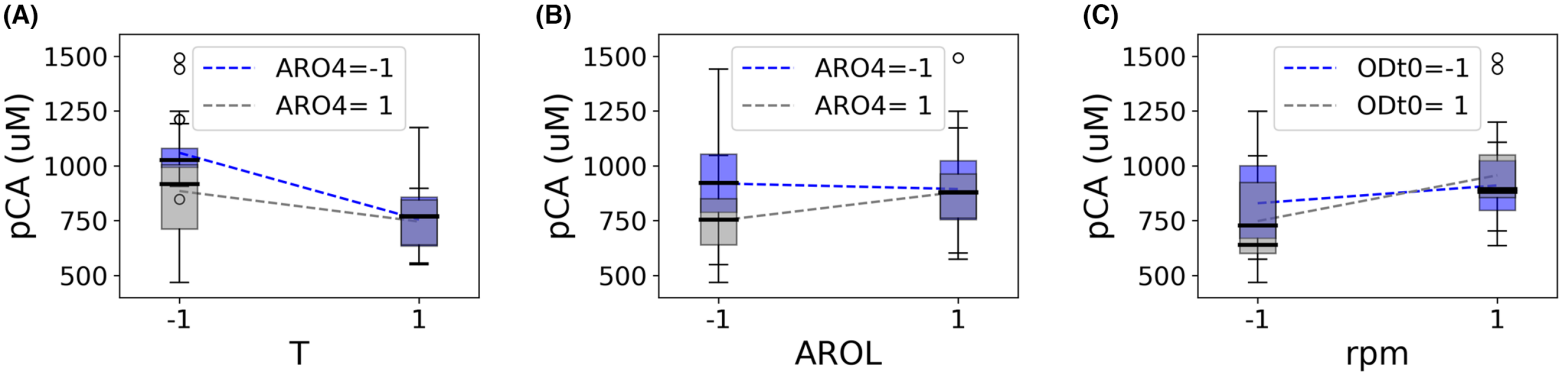
Interaction plots of significant 2‐factor interactions. See Table 2 to identify levels corresponding to −1 and 1. T, temperature; rpm, agitation speed; ODt0, initial cell density.

Interaction graphs were used to understand the relationship between factors involved in significant 2FI and pCA production. Figure 4A shows the interaction between a genetic factor, ARO4, and a process‐related factor, temperature. While at 30°C expression of ARO4 does not affect production, a lower expression results in a higher titre at 20°C. Genetic factors also interact with each other, as an imbalanced expression of low AROL and high ARO4 results in the lowest pCA titre (Figure 4B). Last, a significant interaction between rpm and OD was found since, although fast agitation is always preferred, it has a higher impact in cultures with high initial cell density (Figure 4C).

## DISCUSSION

DoE has been commonly applied to process optimization (Akbarzadeh et al., 2015; Azubuike et al., 2020; Duman‐Özdamar et al., 2022; Lee et al., 2012; Motta Dos Santos et al., 2016; Song et al., 2017; Xu et al., 2008) and strain design (Carbonell et al., 2018; Xu et al., 2017; Young et al., 2018). However, only a few studies consider simultaneous optimization of genetic and environmental factors (Brown et al., 2018; Zhou et al., 2015). Importantly, these studies showed that the interplay between both types of factors must be taken into account simultaneously for the optimization of microbial conversion processes. Our work underscores and strengthens these findings, using pCA production in *S. cerevisiae* as an example. The importance of simultaneous process and strain optimization is highlighted by the T:ARO4 interaction (Figure 4A). If strain selection had been performed at *S. cerevisiae*'s standard growth temperature (30°C), tuning the expression of ARO4 would have been considered irrelevant. However, given that the expression of ARO4 becomes important at lower temperatures, a sub‐optimal strain could have been selected for subsequent process optimization.

The interplay between the strain performance and the bioprocess design is especially important when moving from laboratory‐scale to large‐scale processes. This step‐wise endeavour is time‐consuming, labour‐intensive and expensive. It thus benefits from scale‐down experimentation (Wang et al., 2020). The central paradigm of scaling‐down states that scale‐up will succeed when changes in the cellular environment caused by changes in scale do not influence cell behaviour (Noorman, 2011). Here we show how DoE can identify genetic and process parameters with significant influence on production that should be the focus of the down/up‐scaling plan. We used DoE to understand the effect of 7 process‐related factors (T, rpm, OD, N, pH, Phe and Glu), 4 genetic factors (PAL‐C4H, ARO7, AROL and ARO4) and their interactions on pCA production. Considering two levels per factor, 2048 experiments would be required to find all possible interactions between factors and ensure the identification of the best production conditions. Instead, we performed 92 experiments (4.5% of the total) divided in two consecutive rounds. The first round identified the expression of PAL‐C4H, the addition of Phe and the use of unbuffered media as key variables to ensure high pCA titers. These findings were taken up in a second experimental round for which the optimal pCA production conditions were found: incubation at low temperature and high agitation of a strain with low expression of ARO4. A 38% increased pCA production was obtained in the resolution V round compared to the best experiment in the resolution IV design. Moreover, a 168‐fold variation was measured between the worst and best‐performed experiments.

As indicated, only fourteen of the sixteen strains required for the resolution IV experiments were constructed, so only 28 out of the 32 designed experiments could be performed. Although resolution IV designs with 32 experiments allow the estimation of MEs for up to 16 factors, we considered the impact of 11 factors on production. This granted some redundancy in the design and allowed the estimation of all MEs with 28 out of the 32 designed experiments. However, if more factors had been considered, the construction of all the required strains would have been necessary for the estimation of MEs. This limitation can be solved with the machine learning (ML) analysis of strain libraries generated using one‐pot random transformation (Moreno‐Paz et al., 2023; Zhang et al., 2020). Still, although ML can identify significant factors with an impact on production, quantifying the impact of interactions between factors, critical for bioprocess optimization, is not trivial (Moreno‐Paz et al., 2023). Moreover, the randomization of strains and process conditions, even when mini‐bioreactor systems are used (Janakiraman et al., 2015; Rohe et al., 2012), increases the complexity of creating suitable datasets for ML.

Here we focused on the use of fractional factorial designs to find the optimal production conditions given a design space defined by the selected factors and their levels. To achieve this, a resolution IV design to identify factors with the strongest impact on production and the importance of interactions was employed. These factors were subsequently fixed, and a resolution V design was used to identify the significant interactions. Alternatively, the factors with the most important MEs could have been optimized beyond the original design space. While the pH variable was binary and the use of unbuffered media was recommended, response surface methods could have been employed to optimize the expression of PAL‐C4H and the supplemented Phe concentration (Azubuike et al., 2020; Xu et al., 2017). In this case, higher expression levels and Phe concentrations should have been tested to evaluate the existence of an optimum.

Summarizing, through a systematic evaluation of 11 factors, including genetic modifications and process parameters, we uncovered some of the interplay between genetic and environmental factors in pCA production. Moreover, we demonstrate the power of DoE to provide insights into factor effects and interactions for process optimization. By leveraging the strengths of experimental design and statistical analysis, we provide a framework to find key factors that impact bioprocess performance that could be systematically applied to guide strain design as well as scale‐up/down efforts.

## AUTHOR CONTRIBUTIONS

**Sara Moreno‐Paz:** Conceptualization (equal); data curation (equal); formal analysis (equal); investigation (equal); methodology (equal); visualization (equal); writing – original draft (equal); writing – review and editing (equal). **Rianne van der Hoek:** Conceptualization (equal); data curation (equal); formal analysis (equal); investigation (equal); methodology (equal); writing – original draft (equal); writing – review and editing (equal). **Elif Eliana:** Investigation (equal); writing – review and editing (supporting). **Vitor A. P. Martins dos Santos:** Conceptualization (equal); supervision (equal); writing – review and editing (equal). **Joep Schmitz:** Conceptualization (equal); investigation (equal); methodology (equal); writing – review and editing (equal). **Maria Suarez‐Diez:** Conceptualization (equal); investigation (equal); methodology (equal); writing – review and editing (equal).

## CONFLICT OF INTEREST STATEMENT

RvdH and JS are employed by DSM‐firmenich.

## Supporting information


**Table S1.** Cassettes (A) and sequences (B) used for strain construction.
Table S2. Structure of the gene clusters of constructed strains. (A) Coded values where 1 represents strong promoters and − 1 represents weak promoters. (B) Detailed overview of cassettes introduced in each strain.
Table S3. Resolution IV design, measured p‐coumaric acid (pCA) and predicted production based on linear models including main effects (MEs) or MEs and interactions (2FIs). See table 2 for the correspondence between coded values (−1 and 1) and actual factor levels.
Table S4. Validation experiment of the main effect model derived from resolution IV data.
Table S5. Resolution V design, measured p‐coumaric acid (pCA) and predicted production based on linear models including main effects (MEs) or MEs and interactions (2FIs). See table 2 for the correspondence between coded values (−1 and 1) and actual factor levels.
Table S6. Validation experiment of the main effect model derived from resolution V data.

## References

[mbt214424-bib-0001] Akbarzadeh, A. , Dehnavi, E. , Aghaeepoor, M. & Amani, J. (2015) Optimization of recombinant expression of synthetic bacterial phytase in Pichia pastoris using response surface methodology. Jundishapur Journal of Microbiology, 8(12), 27553. Available from: 10.5812/JJM.27553 PMC4746705 26870311

[mbt214424-bib-0002] Azubuike, C.C. , Edwards, M.G. , Gatehouse, A.M.R. & Howard, T.P. (2020) Applying statistical design of experiments to understanding the effect of growth medium components on cupriavidus necator H16 growth. Applied and Environmental Microbiology, 86(17), e00705‐20. Available from: 10.1128/AEM.00705-20 32561588 PMC7440812

[mbt214424-bib-0003] Braus, G.H. (1991) Aromatic amino acid biosynthesis in the yeast Saccharomyces cerevisiae: a model system for the regulation of a eukaryotic biosynthetic pathway. Microbiological Reviews, 55(3), 349–370. Available from: 10.1128/mmbr.55.3.349-370.1991 1943992 PMC372824

[mbt214424-bib-0004] Brown, S.R. , Staff, M. , Lee, R. , Love, J. , Parker, D.A. , Aves, S.J. et al. (2018) Design of Experiments Methodology to build a multifactorial statistical model describing the metabolic interactions of alcohol dehydrogenase isozymes in the ethanol biosynthetic pathway of the yeast Saccharomyces cerevisiae. ACS Synthetic Biology, 7(7), 1676–1684. Available from: 10.1021/acssynbio.8b00112 29976056

[mbt214424-bib-0005] Carbonell, P. , Jervis, A.J. , Robinson, C.J. , Yan, C. , Dunstan, M. , Swainston, N. et al. (2018) An automated design‐build‐test‐learn pipeline for enhanced microbial production of fine chemicals. Communications Biology, 1(1), 66. Available from: 10.1038/s42003-018-0076-9 30271948 PMC6123781

[mbt214424-bib-0006] Combes, J. , Imatoukene, N. , Couvreur, J. , Godon, B. , Brunissen, F. , Fojcik, C. et al. (2021) Intensification of p‐coumaric acid heterologous production using extractive biphasic fermentation. Bioresource Technology, 337(June), 125436. Available from: 10.1016/j.biortech.2021.125436 34182346

[mbt214424-bib-0007] Combes, J. , Imatoukene, N. , Couvreur, J. , Godon, B. , Fojcik, C. , Allais, F. et al. (2022) An optimized semi‐defined medium for p‐coumaric acid production in extractive fermentation. Process Biochemistry, 122, 357–362. Available from: 10.1016/J.PROCBIO.2022.10.021

[mbt214424-bib-0008] Couto, M.R. , Rodrigues, J.L. & Rodrigues, L.R. (2017) Optimization of fermentation conditions for the production of curcumin by engineered Escherichia coli. Journal of the Royal Society Interface, 14(133), 20170470. Available from: 10.1098/RSIF.2017.0470 28835544 PMC5582133

[mbt214424-bib-0010] de Lorenzo, V. & Couto, J. (2019) The important versus the exciting: reining contradictions in contemporary biotechnology. Microbial Biotechnology, 12(1), 32–34. Available from: 10.1111/1751-7915.13348 30508281 PMC6302708

[mbt214424-bib-0011] Duman‐Özdamar, Z.E. , Martins dos Santos, V.A.P. , Hugenholtz, J. & Suarez‐Diez, M. (2022) Tailoring and optimizing fatty acid production by oleaginous yeasts through the systematic exploration of their physiological fitness. Microbial Cell Factories, 21(1), 1–13. Available from: 10.1186/S12934-022-01956-5/FIGURES/3 36329440 PMC9632096

[mbt214424-bib-0012] Gietz, R.D. , Schiestl, R.H. , Willems, A.R. & Woods, R.A. (1995) Studies on the transformation of intact yeast cells by the LiAc/SS‐DNA/PEG procedure. Yeast, 11(4), 355–360. Available from: 10.1002/YEA.320110408 7785336

[mbt214424-bib-0013] Gilman, J. , Walls, L. , Bandiera, L. & Menolascina, F. (2021) Statistical Design of Experiments for synthetic biology. ACS Synthetic Biology, 10(1), 1–18. Available from: 10.1021/acssynbio.0c00385 33406821

[mbt214424-bib-0014] Grömping, U. (2014) R package FrF2 for creating and analyzing fractional factorial 2‐level designs. Journal of Statistical Software, 56(1), 1–56. Available from: 10.18637/JSS.V056.I01

[mbt214424-bib-0015] Janakiraman, V. , Kwiatkowski, C. , Kshirsagar, R. , Ryll, T. & Huang, Y.M. (2015) Application of high‐throughput mini‐bioreactor system for systematic scale‐down modeling, process characterization, and control strategy development. Biotechnology Progress, 31(6), 1623–1632. Available from: 10.1002/BTPR.2162 26317495

[mbt214424-bib-0016] Jendresen, C.B. , Stahlhut, S.G. , Li, M. , Gaspar, P. , Siedler, S. , Förster, J. et al. (2015) Highly active and specific tyrosine ammonia‐lyases from diverse origins enable enhanced production of aromatic compounds in bacteria and Saccharomyces cerevisiae. Applied and Environmental Microbiology, 81(13), 4458–4476. Available from: 10.1128/AEM.00405-15/SUPPL_FILE/ZAM999116361SO1.PDF 25911487 PMC4475877

[mbt214424-bib-0017] Jeschek, M. , Gerngross, D. & Panke, S. (2017) Combinatorial pathway optimization for streamlined metabolic engineering. Current Opinion in Biotechnology, 47, 142–151. Available from: 10.1016/J.COPBIO.2017.06.014 28750202

[mbt214424-bib-0018] Kim, G.B. , Choi, S.Y. , Cho, I.J. , Ahn, D.H. & Lee, S.Y. (2023) Metabolic engineering for sustainability and health. Trends in Biotechnology, 41(3), 425–451. Available from: 10.1016/J.TIBTECH.2022.12.014 36635195

[mbt214424-bib-0019] Koopman, F. , Beekwilder, J. , Crimi, B. , van Houwelingen, A. , Hall, R.D. , Bosch, D. et al. (2012) De novo production of the flavonoid naringenin in engineered Saccharomyces cerevisiae. Microbial Cell Factories, 11(1), 1–15. Available from: 10.1186/1475-2859-11-155/TABLES/3 23216753 PMC3539886

[mbt214424-bib-0020] Lawson, J. (2014) Design and analysis of experiments with R. Boca Raton, FL: CRC press.

[mbt214424-bib-0021] Lee, Y.J. , Kim, H.J. , Gao, W. , Chung, C.H. & Lee, J.W. (2012) Statistical optimization for production of carboxymethylcellulase of bacillus amyloliquefaciens DL‐3 by a recombinant Escherichia coli JM109/DL‐3 from rice bran using response surface method. Biotechnology and Bioprocess Engineering, 17(2), 227–235. Available from: 10.1007/S12257-011-0258-5/METRICS

[mbt214424-bib-0022] Liu, Q. , Yu, T. , Li, X. , Chen, Y. , Campbell, K. , Nielsen, J. et al. (2019) Rewiring carbon metabolism in yeast for high level production of aromatic chemicals. Nature Communications, 10(1), 1–13. Available from: 10.1038/s41467-019-12961-5 PMC6823513 31672987

[mbt214424-bib-0023] Moreno‐Paz, S. , van der Hoek, R. , Eliana, E. , Zwartjens, P. , Gosiewska, S. , Martins dos Santos, V.A.P. et al. (2023) Machine learning‐guided optimization of p‐coumaric acid production in yeast. bioRxiv. Available from: 10.1101/2023.11.27.568789 PMC11036487 38545878

[mbt214424-bib-0024] Motta dos Santos, L. , Coutte, F. , Ravallec, R. , Dhulster, P. , Tournier‐Couturier, L. & Jacques, P. (2016) An improvement of surfactin production by B. Subtilis BBG131 using design of experiments in microbioreactors and continuous process in bubbleless membrane bioreactor. Bioresource Technology, 218, 944–952. Available from: 10.1016/J.BIORTECH.2016.07.053 27447921

[mbt214424-bib-0025] NCBI . (2024) *Saccharomyces cerevisiae CEN.PK113‐7D contig163, whole genome shotgun*–*Nucleotide*–*NCBI* . Available from: https://www.ncbi.nlm.nih.gov/nuccore/AEHG01000256 [Accessed 8th January 2024].

[mbt214424-bib-0026] Noorman, H. (2011) An industrial perspective on bioreactor scale‐down: what we can learn from combined large‐scale bioprocess and model fluid studies. Biotechnology Journal, 6(8), 934–943. Available from: 10.1002/BIOT.201000406 21695785

[mbt214424-bib-0027] Prins, R.C. & Billerbeck, S. (2021) A buffered media system for yeast batch culture growth. BMC Microbiology, 21(1), 1–9. Available from: 10.1186/s12866-021-02191-5 33892647 PMC8063419

[mbt214424-bib-0028] Rodriguez, A. , Kildegaard, K.R. , Li, M. , Borodina, I. & Nielsen, J. (2015) Establishment of a yeast platform strain for production of p‐coumaric acid through metabolic engineering of aromatic amino acid biosynthesis. Metabolic Engineering, 31, 181–188. Available from: 10.1016/j.ymben.2015.08.003 26292030

[mbt214424-bib-0029] Rohe, P. , Venkanna, D. , Kleine, B. , Freudl, R. & Oldiges, M. (2012) An automated workflow for enhancing microbial bioprocess optimization on a novel microbioreactor platform. Microbial Cell Factories, 11(1), 1–14. Available from: 10.1186/1475-2859-11-144/FIGURES/6 23113930 PMC3526558

[mbt214424-bib-0030] So, K.K. , Le, N.M.T. , Nguyen, N.L. & Kim, D.H. (2023) Improving expression and assembly of difficult‐to‐express heterologous proteins in Saccharomyces cerevisiae by culturing at a sub‐physiological temperature. Microbial Cell Factories, 22(1), 55. Available from: 10.1186/S12934-023-02065-7/FIGURES/9 36959657 PMC10035479

[mbt214424-bib-0031] Song, T.Q. , Ding, M.Z. , Zhai, F. , Liu, D. , Liu, H. , Xiao, W.H. et al. (2017) Engineering Saccharomyces cerevisiae for geranylgeraniol overproduction by combinatorial design. Scientific Reports, 7(1), 1–11. Available from: 10.1038/s41598-017-15005-4 29118396 PMC5678108

[mbt214424-bib-0032] Trantas, E. , Panopoulos, N. & Ververidis, F. (2009) Metabolic engineering of the complete pathway leading to heterologous biosynthesis of various flavonoids and stilbenoids in Saccharomyces cerevisiae. Metabolic Engineering, 11(6), 355–366. Available from: 10.1016/J.YMBEN.2009.07.004 19631278

[mbt214424-bib-0033] Verwaal, R. , Buiting‐Wiessenhaan, N. , Dalhuijsen, S. & Roubos, J.A. (2018) CRISPR/Cpf1 enables fast and simple genome editing of Saccharomyces cerevisiae. Yeast, 35(2), 201–211. Available from: 10.1002/YEA.3278 28886218 PMC5836994

[mbt214424-bib-0034] Wang, G. , Haringa, C. , Noorman, H. , Chu, J. & Zhuang, Y. (2020) Developing a computational framework to advance bioprocess scale‐up. Trends in Biotechnology, 38(8), 846–856. Available from: 10.1016/j.tibtech.2020.01.009 32493657

[mbt214424-bib-0035] Xu, P. , Ding, Z.Y. , Qian, Z. , Zhao, C.X. & Zhang, K.C. (2008) Improved production of mycelial biomass and ganoderic acid by submerged culture of Ganoderma lucidum SB97 using complex media. Enzyme and Microbial Technology, 42(4), 325–331. Available from: 10.1016/j.enzmictec.2007.10.016

[mbt214424-bib-0036] Xu, P. , Rizzoni, E.A. , Sul, S.Y. & Stephanopoulos, G. (2017) Improving metabolic pathway efficiency by statistical model‐based multivariate regulatory metabolic engineering. ACS Synthetic Biology, 6(1), 148–158. Available from: 10.1021/acssynbio.6b00187 27490704

[mbt214424-bib-0037] Young, E.M. , Zhao, Z. , Gielesen, B.E.M. , Wu, L. , Benjamin Gordon, D. , Roubos, J.A. et al. (2018) Iterative algorithm‐guided design of massive strain libraries, applied to itaconic acid production in yeast. Metabolic Engineering, 48, 33–43. Available from: 10.1016/j.ymben.2018.05.002 29753070

[mbt214424-bib-0038] Zhang, J. , Petersen, S.D. , Radivojevic, T. , Ramirez, A. , Pérez‐Manríquez, A. , Abeliuk, E. et al. (2020) Combining mechanistic and machine learning models for predictive engineering and optimization of tryptophan metabolism. Nature Communications, 11(1), 1–13. Available from: 10.1038/s41467-020-17910-1 PMC7519671 32978375

[mbt214424-bib-0039] Zhou, H. , Vonk, B. , Roubos, J.A. , Bovenberg, R.A.L. & Voigt, C.A. (2015) Algorithmic co‐optimization of genetic constructs and growth conditions: application to 6‐ACA, a potential nylon‐6 precursor. Nucleic Acids Research, 43(21), gkv1071–gk10570. Available from: 10.1093/nar/gkv1071 PMC4666358 26519464

